# Mother-to-Child Signaling through Breast Milk Biomolecules

**DOI:** 10.3390/biom11121743

**Published:** 2021-11-23

**Authors:** Tamás Röszer

**Affiliations:** Institute of Neurobiology, Faculty of Science, Ulm University, 89081 Ulm, Germany; tamas.roeszer@uni-ulm.de

Breastfeeding—or lactation—is a unique and defining reproductive trait of mammals that nourishes offspring by supplying nutrient-rich breast milk. In addition to being the best source of nutrition for the infant, breast milk confers important and lasting infant and maternal health benefits, including the proper development of the immune system in the infant and the programming of endocrine and metabolic functions (Figure 1). The biomolecules of the breast milk drive a fascinating mother-to-child signaling network through breastfeeding. In turn, infant tissues express specific receptors for breast milk biomolecules, and harness active bioconversion processes to metabolize breast milk to gain energy and to elaborate signal molecules (e.g., lipid mediators). Breast milk antibodies, cytokines, and other immune molecules ensure disease immunity, protect the developing intestinal barrier from inflammation, and reduce the risk of chronic inflammatory diseases. Moreover, the hormones, cytokines, lipids, vitamins, and oligosaccharides of the breast milk control organ differentiation, thermogenesis, and cognitive development, influence food preference in childhood, and initiate the growth of normal microbial flora [1,2,3,4,5,6,7,8,9,10,11]. All of this can be lost with insufficient breastfeeding, which promotes childhood obesity and increases the risk of inflammatory diseases and diabetes later in life [1,5,7,8,9,12,13,14].

Worryingly, rates of breastfeeding are declining dramatically, which, consequentially, is likely to have a negative impact on infant and adolescent health [6,8]. Understanding the health impact of breast milk and formula milk biomolecules is, therefore, timely and important. This Special Issue of *Biomolecules* brings together a collection of articles addressing relevant aspects of the role of breast milk biomolecules as signal transmitters between a mother and child.

Lipid metabolism in infants is orchestrated by breast milk lipid signals, and the declining breastfeeding rates are paralleled by an alarming increase in childhood obesity, which, at least in part, may have its roots in impaired metabolic control by breast milk lipid signals. Moreover, there is a shared evolutionary history between lactation and the unique lipid-metabolizing capabilities of the infant adipose tissue in mammals [15].

Melnik et al. provide an extensive review of the microRNA cargo of breast milk extracellular vesicles (EVs) [16], and its role in modulating the key immune, endocrine, and metabolic effects of breastfeeding. This includes the suppression of nuclear factor-κB signaling and protection from necrotizing enterocolitis, in addition to promoting the thermogenic fat development and epigenetic programming of several organs including the liver, thymus, brain, and pancreatic islets.

Vahkal et al. have written an important and timely review on the proteomic analysis of human breast milk EVs. While the biological impact of communication from mother to a breastfed child via breast milk EVs is now established, the experimental characterization and functional analysis of breast milk EVs are not simple tasks. Vahkal et al. provide a methodological workflow with the goal of producing effective and reproducible analysis of the human breast milk EV proteome [17].

A paper by Bae et al. reviews the role of breast milk in the control of angiogenesis, a key element in normal organ development [18]. They describe how human oligosaccharides, and sialylated human milk oligosaccharides in particular, are key maternal signals in the control of angiogenesis by modulating vascular endothelial growth factor signaling.

A richer understanding of the health impact of the repertoire of breast milk biomolecules, including lipids, RNA species, proteins of extracellular vesicles, and oligosaccharides, should have far-reaching societal benefits due to their fundamental roles in determining postnatal development. As the Guest Editor of this Special Issue, I would like to thank the contributors for their efforts in preparing the papers, the referees for their constructive feedback, and the editorial and production teams of *Biomolecules*. I hope that this collection of articles will be useful for teaching and research, will stimulate discussion, open up new avenues of research in this growing area, and be an important contribution to the existing literature.

## Figures and Tables

**Figure 1:**
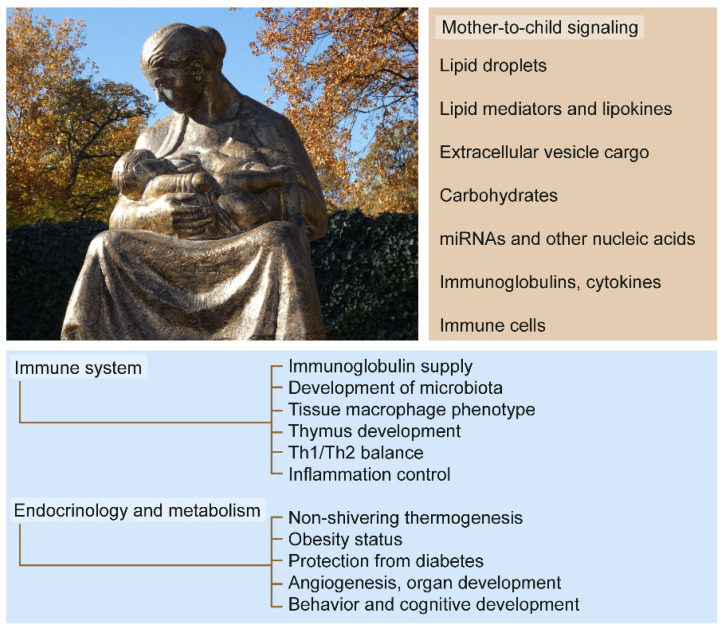
Mother-to-child signaling through breastfeeding. Breastfeeding provides maternal biomolecules such as lipids (e.g., alkylglycerols and lipokines), microRNA species, milk-specific oligosaccharides (e.g., sialylated human milk oligosaccharides), and a rich protein cargo of extracellular vesicles. Infant tissues express receptors for maternal biomolecules. Breast milk molecules control immune maturation, organ development, and the endocrine health of the infant. Ultimately, breastfeeding shapes energy homeostasis and functions as an early life determinant of metabolic health. Image: “Breastfeeding mother” sculpture by Ferenc Medgyessy (1881–1958), in a public park in Debrecen, Hungary. Photo taken by Author.

## Data Availability

Not applicable.
